# Dietary Arginine Sets the Pace of MHC Class I Translation: A Nutrient Lever on Tumour Immunogenicity

**DOI:** 10.1002/mco2.71121

**Published:** 2026-10-27

**Authors:** Mohammad Asim

**Affiliations:** ^1^ Molecular Oncotherapeutics Laboratory Department of Clinical and Experimental Medicine School of Biosciences University of Surrey Guildford UK

**Keywords:** arginine, ASS1, codon‐biased translation, MHC class I, transfer RNA, tumor immunogenicity

1

In a recent study published in Cell, Wu and colleagues reported that extracellular arginine sets the rate at which cells translate major histocompatibility complex class I (MHC class I), because that coding sequence is rich in arginine codons and scarcity depletes the charged arginyl‐transfer RNAs (tRNAs) decoding them [1]. Restoring arginine by diet or by deleting myeloid arginase rebuilt surface MHC class I, reduced colorectal tumour burden and improved antiviral immunity; a cheap nutrient thus becomes a candidate immunotherapeutic variable.

Arginine falls further than most amino acids in tumours, and in infected tissue because myeloid cells secrete arginase, which degrades it. That scarcity has been read mainly as a brake on the T cells themselves, which require arginine to proliferate and to kill. Wu et al. locate an earlier lesion in the capacity of the tumour cell to build the surface complex that displays fragments of its own proteins to cytotoxic CD8‐positive T cells.

The control point is translational elongation. Each codon of a messenger RNA (mRNA) is matched to its amino acid by a charged tRNA, and arginine is served by five isoacceptor tRNAs, of which arginine starvation destabilises four while sparing the fifth, which reads the codon AGG. Because the MHC class I coding sequence carries an unusually high density of arginine codons, the shortfall falls heavily on that transcript. Ribosome profiling showed increased ribosome occupancy at arginine codons across the MHC class I transcript, consistent with pausing, and full‐length MHC class I protein fell; whether the paused ribosomes release truncated products was not directly tested. Two alternative explanations were excluded. MHC class I mRNA increased rather than decreased, so reduced transcript abundance does not account for the loss of protein. The protein also became more stable rather than less, and inhibiting proteasomal and lysosomal degradation did not restore it, so accelerated turnover is insufficient. Depleting arginyl‐tRNA synthetase, the enzyme that charges arginine onto its tRNA, reproduced most of the effect, which separates the demand for arginine as a substrate for protein synthesis from its other metabolic uses (Figure 1).

**FIGURE 1:**
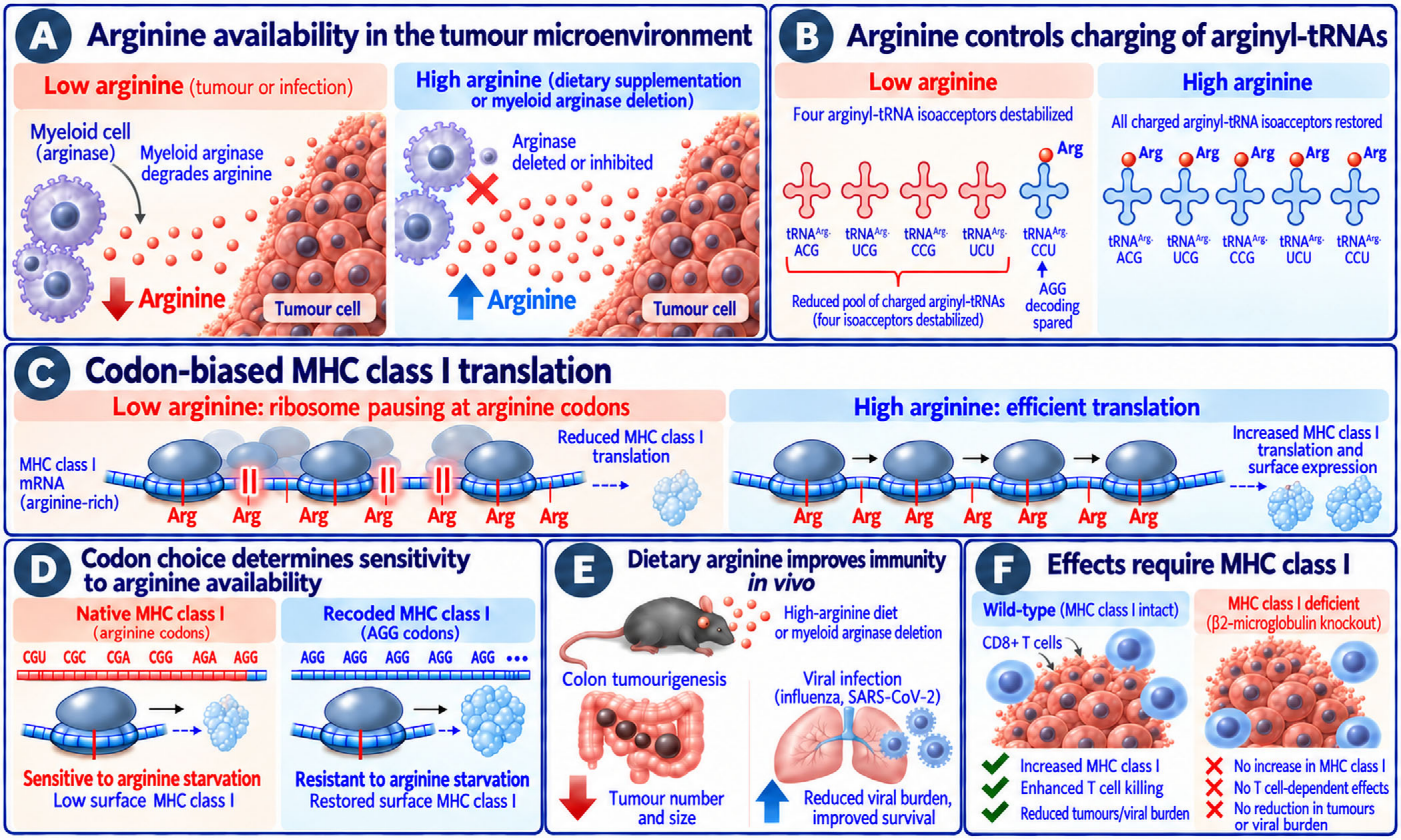
Arginine availability regulates codon‐biased MHC class I translation and immune recognition. (A) In arginine‐poor tumour or infected tissue, myeloid‐cell arginase degrades extracellular arginine, whereas dietary arginine supplementation or myeloid arginase deletion increases local arginine availability. (B) Arginine limitation reduces the pool of charged arginyl‐tRNAs; four arginyl‐tRNA isoacceptors are destabilised, while the AGG‐reading tRNA is relatively spared. Restoring arginine replenishes charged arginyl‐tRNAs. (C) Because MHC class I mRNA is enriched in arginine codons, reduced arginyl‐tRNA availability causes ribosome pausing at arginine codons and lowers MHC class I protein production and surface expression. Increasing arginine availability relieves this translational bottleneck and restores MHC class I expression. (D) Codon choice determines sensitivity to arginine limitation. Native MHC class I sequences containing pause‐prone arginine codons remain sensitive to arginine starvation, whereas synonymous recoding to the relatively spared AGG codon maintains translation and surface MHC class I expression without altering the encoded amino‐acid sequence. (E) In mouse models, a high‐arginine diet or deletion of myeloid arginase increased MHC class I expression and was associated with reduced tumour burden and improved outcomes after respiratory viral infection, including reduced viral burden and improved survival. (F) These effects depend on intact MHC class I antigen presentation: in MHC class I‐deficient mice lacking β2‐microglobulin, arginine supplementation did not increase MHC class I‐dependent T‐cell activity or reproduce the reductions in tumour or viral burden observed in MHC class I‐intact animals. Together, these findings identify extracellular arginine availability as a regulator of codon‐dependent MHC class I translation and, consequently, immune recognition. MHC, major histocompatibility complex; β2‐microglobulin, beta‐2‐microglobulin.

A single genetic experiment lifts this above correlation. Recoding the arginine codons of an MHC class I gene so that all used the spared AGG codon, without altering a single amino acid, made expression largely resistant to arginine starvation, restored surface antigen and returned efficient killing by antigen‐specific T cells. A natural counterpart already existed in the RKO colorectal line, which carries two sequences at a pause‐prone position of one MHC class I gene: the arginine‐encoding copy stalled while the serine‐encoding copy, in the same cell, did not. Codon usage, independently of the encoded protein, determines translational efficiency, so synonymous mutations long treated as silent can contribute to whether a tumour is visible to the immune system.

The animal work supplies the physiological consequence. In a chemically induced model of colon cancer, deleting arginase from myeloid cells raised intratumoral arginine, increased MHC class I and reduced tumour number and size; a high‐arginine diet changed these outcomes in the same direction and a low‐arginine diet in the opposite direction. Dietary manipulation also altered weight loss, lung viral burden and survival after influenza and SARS‐CoV‐2 infection. These benefits were lost in mice lacking beta‐2‐microglobulin, the invariant chain required for MHC class I to fold and reach the cell surface, which demonstrates that MHC class I is directly required for the observed effects rather than merely accompanying them.

These observations extend a decade of ground‐breaking work from the Tavazoie laboratory establishing that tRNA supply, rather than transcript abundance alone, determines which proteins a cancer cell makes. The Tavazoie laboratory previously showed that arginine limitation can drive mutational evolution away from arginine codons, reducing translational bottlenecks during arginine starvation [2]. Placing MHC class I inside that framework converts codon‐biased translation from an account of tumour cell‐autonomous growth into a mechanism of immune evasion. Whether the same evolutionary pressure preferentially protects proliferative genes while leaving antigen‐presentation genes behind is a question that existing sequence data could answer.

The clinical inference is less direct than it first appears, because arginine has been manipulated in both directions in patients with results that depend on tumour type, on the agent it is combined with, and on whether depletion is sustained. Tumors that silence argininosuccinate synthetase 1 (ASS1) cannot synthesise arginine and depend on import, and in non‐epithelioid pleural mesothelioma the addition of pegylated arginine deiminase (pegargiminase) to first‐line chemotherapy improved median overall survival from 7.7 to 9.3 months (hazard ratio 0.71, 95% CI 0.55–0.93; *p* = 0.02) and raised 36‐month survival from 3.3% to 11.9% [3]. The same agent given as monotherapy to 635 patients with advanced hepatocellular carcinoma produced no survival benefit (7.8 vs. 7.4 months; *p* = 0.88), although patients in whom depletion was sustained beyond seven weeks fared better [4]. Moving in the opposite direction has been no more straightforward. Pharmacological arginase inhibition with INCB001158, given alone or with pembrolizumab to 260 patients across several tumour types, achieved measurable target engagement but response rates that did not exceed those expected for the tumour types treated [5]. Increasing systemic arginine is therefore not equivalent to immune activation. Arginine also feeds nitric oxide and polyamine pathways whose immune effects are cell‐ and context‐dependent, so increasing systemic arginine cannot be assumed to favour antitumour immunity.

Wu et al. help explain why these trials diverge. Depletion attacks a metabolic dependency in tumours that cannot synthesise arginine, whereas restoration relieves a translational deficit in tumours that retain arginine‐synthesis capacity but sit in surroundings that suppress antigen display. The two strategies are aimed at different patients rather than in genuine contradiction, and ASS1 status, already assessed routinely by immunohistochemistry, is one variable that separates them. A second constraint narrows the eligible population. Much of the MHC class I loss seen in human tumours is structural rather than translational: allele‐specific loss of human leukocyte antigen (HLA) haplotypes is common in non‐small‐cell lung cancer, and beta‐2‐microglobulin mutation or deletion recurs in patients progressing on checkpoint blockade. No dietary intervention restores a deleted gene. The patients worth testing are those whose antigen‐presentation machinery is translationally suppressed but structurally intact.

Two experiments follow. Because systemic arginine depletion could lower MHC class I in arginine‐responsive cells with intact antigen‐presenting machinery, pegargiminase may antagonise concurrent checkpoint blockade in ASS1‐retaining tumours, which can be examined in banked trial material. And because recoding to AGG protects expression, MHC transgenes, engineered T‐cell receptors, and vaccine antigens engineered the same way could be tested to preserve expression in arginine‐poor tissue where conventional designs fail. Arginine is inexpensive, oral, and safe at nutritional doses; MHC class I expression is measurable; ASS1 staining is routine. Wu and colleagues have followed one amino acid from tRNA charging to a synonymous‐codon rescue of antitumor immunity in vivo, and the reagents needed to test the idea in a stratified population already exist.

## Author Contributions

Mohammad Asim conceived, wrote, and revised the manuscript and prepared the figure. The author has read and approved the final manuscript.

## Funding

The authors have nothing to report.

## Ethics Statement

The authors have nothing to report.

## Conflicts of Interest

The author declares no conflicts of interest. The author was not involved in the peer review of the highlighted study and has no collaborative association with its authors.

## Data Availability

The author has nothing to report.
